# Multidimensional Impact of Dupilumab on Chronic Rhinosinusitis with Nasal Polyps: A Complete Health Technology Assessment of Clinical, Economic, and Non-Clinical Domains

**DOI:** 10.3390/jpm14040347

**Published:** 2024-03-27

**Authors:** Ignazio La Mantia, Giancarlo Ottaviano, Martina Ragusa, Matteo Trimarchi, Emanuela Foglia, Fabrizio Schettini, Daniele Bellavia, Elena Cantone

**Affiliations:** 1 Department of Medical and Surgical Sciences and Advanced Technologies “GF Ingrassia”, ENT Section, University of Catania, 95100 Catania, Italy; 2 Department of Neurosciences, Otolaryngology Section, University of Padova, 35122 Padova, Italy; 3 Operative Unit of Otolaryngology, ASP Messina, Taormina Hospital (ME), 98039 Taormina, Italy; 4 Faculty of Biomedical Sciences and Lugano Regional Hospital, University of Italian Switzerland, 6900 Lugano, Switzerland; 5 Healthcare Datascience LAB, LIUC—Carlo Cattaneo University, 21053 Castellanza, Italy; 6 Department of Neuroscience, Reproductive and Odontostomatological Sciences-ENT Section, University of Naples 29 Federico II, 80131 Naples, Italy

**Keywords:** CRSwNP, nasal polyposis, health technology assessment, HTA, economic assessment, cost per responder

## Abstract

Chronic RhinoSinusitis with Nasal Polyps (CRSwNP) represents a condition mainly caused by the type 2 inflammation presence and marked by the existence of polyps within the nasal and paranasal sinuses. The standard of care includes intranasal steroids, additional burst of systemic steroids, if needed, and surgery. However, recurrence is common, especially among patients with comorbid type 2 inflammatory diseases. Recently, biological drugs, addressing the underlying cause of the disease, have been approved in Italy (dupilumab, omalizumab, and mepolizumab). A Health Technology Assessment was conducted to define multidimensional impact, assuming Italian NHS perspective and a 12-month time horizon. The EUnetHTA Core Model was deployed, using the following methods to analyze the domains: (i) literature evidence; (ii) administration of semi-structured questionnaires to 17 healthcare professionals; (iii) health economics tools to define the economic sustainability for the system. Evidence from NMA and ITC showed a more favorable safety profile and better efficacy for dupilumab compared with alternative biologics. All the analyses, synthesizing cost and efficacy measures, showed that dupilumab is the preferable alternative. Specifically, the cost per responder analysis for dupilumab, exhibiting a 67.0% response rate at Week 52, is notably economical at 14,209EUR per responder. This presents a more economical profile compared with the cost per responder for omalizumab (36.2% response rate) at 24,999EUR and mepolizumab (28.5% response rate) at 31,863EUR. These results underscore dupilumab’s potential, not merely in terms of clinical outcomes, but also in terms of economic rationality, thereby solidifying its status as a valid and preferrable alternative in the management of CRSwNP, in the context of the Italian NHS.

## 1. Introduction

Chronic rhinosinusitis (CRS) is a prevalent condition in Italy, affecting approximately 79,500 individuals annually, with significant social and economic repercussions [1]. CRS, impacting 5–12% of the general population, is categorized into two phenotypes based on the presence of nasal polyps: Chronic RhinoSinusitis without Nasal Polyps (CRSsNP) and Chronic RhinoSinusitis with Nasal Polyps (CRSwNP) [2]. CRSwNP is often associated with type 2 inflammation, characterized by elevated type 2 cytokines (IL-4, IL-13, IL-5) and increased tissue and serum IgE levels, and is frequently accompanied by bilateral nasal polyps and chronic paranasal sinus inflammation [2,3]. These symptoms severely affect patients’ quality of life and productivity [4,5]. Additionally, CRSwNP patients often experience comorbid type 2 inflammatory conditions such as asthma and allergic rhinitis [6,7], adding to the overall disease burden [8,9]. Nasal saline irrigation and intranasal corticosteroids (INCS) are the standard treatments for CRSwNP, with oral systemic corticosteroids (OCS) and sino-nasal surgeries reserved for more severe cases [10,11]. However, disease recurrence following surgery is common. Dupilumab, a human monoclonal antibody targeting IL-4 and IL-13, has emerged as the first biologic approved in Italy for CRSwNP, offering significant experience in real-life settings compared to other biologics like omalizumab and mepolizumab [12]. While head-to-head (H2H) studies are lacking, three studies classified as independent indirect treatment comparisons (ITCs) or network meta-analyses (NMAs) have been published [12,13,14], in addition to the efficacy demonstrated in randomized clinical trials (RCTs) [15,16,17]. The absence of direct comparative studies and specific biomarkers for biologic selection underscores the importance of the indirect treatments’ comparisons and of the network metanalysis [12,13,14]. The results of these studies suggest that among comparable patient populations, dupilumab is associated with significantly greater improvements for all outcomes studied compared with the other biologics.

While the effectiveness and safety of current treatments are well-documented, an in-depth analysis of their economic and non-clinical impact is often missing. Non-clinical domains potentially significantly impact the healthcare organizations taking in charge CRSwNP patients, and in line with the recent HTA European Regulation, require specific attention on these topics to investigate the peculiarities in the local contexts (Regulation (EU) 2021/2282) [18]. Such an analysis is crucial for understanding the broader implications of therapeutic choices, particularly in terms of economic and organizational aspects. This study aims to provide a comprehensive Health Technology Assessment (HTA) of CRSwNP treatments, focusing on the economic and non-clinical impacts of biologics. The HTA’s systematic and multidisciplinary approach allows for a thorough evaluation of health technologies and interventions, considering not only direct consequences but also organizational, social, and ethical dimensions [19]. The primary research question posed in this study was as follows: “What is the multidimensional impact of biologic treatments on chronic rhinosinusitis with nasal polyposis, in terms of resources utilization, clinical outcomes, and non-clinical factors, both from a quantitative and a qualitative perspective?” Addressing this research question is pivotal in guiding healthcare decision-making processes on the potential clinical and economic benefits of dupilumab compared to the other biologic therapeutic alternatives [20].

## 2. Materials and Methods

This study adopts the perspective of the Italian National Healthcare Service (NHS); the parameters were selected, and the analyses were conducted considering a 12-month time horizon for the CRSwNP patients’ treatment and follow-up. The initial phase encompassed a literature review, geared towards extracting key information on CRSwNP, focusing on context, efficacy, and safety profiles of the various treatments under assessment. Subsequently, structured interviews were conducted with healthcare professionals specialized in treating CRSwNP. Moreover, typical health economics methodologies, primarily quantitative, were employed for the economic analysis. The study was structured as follows.

(i) Definition of the target population
(ii) Selection of technologies to assess
(iii) Selection of studies and assessment of the quality of literature evidence [21]
(iv) Assessment of the dimensions

### 2.1. Target Population

To estimate the population eligible for treatment, the prevalence of CRSwNP [22,23] was applied to the Italian adult population residents in 2022 (ISTAT, 2023). Therefore, a funnel of inclusion criteria was applied, estimating the number of Italian patients with severe CRSwNP for whom standards of care were not effective.

### 2.2. Selection of Technologies under Assessment

Therapeutic alternatives for CRSwNP were examined, including biologic therapies, surgical intervention, and corticosteroid treatment.

Thus, the following alternatives were assessed in the HTA analysis.

1. Biologic dupilumab: a human monoclonal antibody that targets and blocks the effects of IL-4 and IL-13 cytokines, key drivers of type 2 inflammation.
2. Biologic omalizumab: a humanized monoclonal antibody that selectively binds to human immunoglobulin E (IgE), as noted by AIFA (Italian Drug Agency) (2020).
3. Biologic mepolizumab: a humanized monoclonal antibody that acts by inhibiting circulating interleukin-5 (IL-5), crucial for the maturation and activity of eosinophils, according to AIFA (2020).

### 2.3. Screening and Selection of the Studies: PICO Definition

Once the research question, the target population of reference, and the technologies to assess were defined, the PICO (Patient/Population, Intervention, Comparator, Outcome) framework was determined and applied as follows.

- Population: adults with severe CRSwNP for whom systemic corticosteroid therapy and/or surgery do not provide adequate disease control.
- Intervention: the use of the biologic drug dupilumab.
- Comparisons: biologic drugs omalizumab and mepolizumab.
- Outcome measures:
  ○ efficacy, measured using the SNOT-22 scale (Sino-Nasal Outcome Test 22 items); NPS scale (Nasal Polyp Score), VAS (Visual Analogue Scale), UPSIT scale (University of Pennsylvania Smell Identification Test);
  ○ safety, evaluating severe, mild, and moderate adverse events, and discontinuation or interruption of treatment, due to adverse events;
  ○ economic, analyzing healthcare costs associated with different biologics, and a cost per responder analysis proving evidence on the comparative economic and clinical efficacy of alternative biologic treatments in CRSwNP.

After defining the PICO, an analysis of national and international evidence was carried out through a narrative literature search using the main databases (Medline/Pubmed, Embase, and Cochrane Database) [24]. In accordance with the PRISMA (Preferred Reporting Items for Systematic Reviews and Meta-Analyses) guidelines, the most relevant publications were identified and selected for analysis and validation, considering the following search strategy [25]:

(Nasal AND polyps OR nasal AND polyposis OR crswnp OR chronic AND rhinosinusitis) AND (dupilumab OR dupixent OR omalizumab OR mepolizumab OR biologics OR fess OR ess OR endoscopic AND sinus AND surgery OR surgery OR ocs OR corticosteroid OR steroid) AND (efficacy OR effectiveness OR snot22 OR snot-22 OR nps OR vas OR upsit OR quality AND of AND life OR comorbidities OR asthma OR inflammatory AND disease OR type-2 OR cross-coverage OR epidemiology OR prevalence OR incidence OR population OR safety OR adverse AND events OR complication OR reactions) AND (LIMIT-TO (PUBYEAR, 2023) OR LIMIT-TO (PUBYEAR, 2022) OR LIMIT-TO (PUBYEAR, 2021) OR LIMIT-TO (PUBYEAR, 2020) OR LIMIT-TO (PUBYEAR, 2019) OR LIMIT-TO (PUBYEAR, 2018) OR LIMIT-TO (PUBYEAR, 2017) OR LIMIT-TO (PUBYEAR, 2016) OR LIMIT-TO (PUBYEAR, 2015)) AND (LIMIT-TO (DOCTYPE, “ar”) OR LIMIT-TO (DOCTYPE, “re”)).

The strategy employed for the literature review did not exclusively limit the inquiry to studies published by authors with affiliations in Italy. This approach was taken to enrich the understanding of the context with relevant international literature. Despite the focus on Italy, the search strategy was designed to capture significant contributions from both national and international sources. Due to the specific search criteria used, a manual selection of articles was necessary to ensure relevance to the Italian context, incorporating international studies that provide valuable insights. This selection process aimed at maintaining a comprehensive understanding of the subject matter, transcending national boundaries.

Subsequently, the quality of the retrieved literature was assessed by five experts, using the JADAD scale for RCTs and the Newcastle–Ottawa Scale for the other studies [26,27].

### 2.4. Multidimensional Assessment

To achieve the primary objective of the proposed study, a complete Health Technology Assessment was performed, based on the EUnetHTA Core Model implementation. The model’s clinical and non-clinical domains were analyzed, in particular: relevance of the pathology, technical characteristics of the alternatives, safety, efficacy, economic–financial impact, equity dimension, organizational impact, social aspects, and legal aspects. 

In HTA evaluations, clinical domains can be assessed through an analysis of scientific literature evidence. However, for non-clinical domains, it is appropriate and often necessary to employ qualitative research methods and rely on the expertise of specialists in the specific field. This approach is vital for exploring areas where clinical evidence may be limited or absent, allowing for a deeper understanding of complex or controversial issues. Qualitative research, often used in social sciences, is characterized by high flexibility, enabling a thorough exploration of attitudes, experiences, and intentions [28]. It generates a broad range of ideas and opinions regarding specific problems and issues, considering different perspectives among stakeholder groups. These methods, as briefly outlined in the previous section, attempt to bridge the gaps and knowledge gaps emerging from scientific evidence [29]. It is important to underline that HTA produces an estimation of potential impact, and it necessitates subsequent consolidation through real-world evidence collection or updates based on the lifecycle of alternative technologies [30].

In order to investigate each of the EUnetHTA domains, a structured collection of expert opinions was carried out through interviews with healthcare professionals in Italy, involving 17 ENT clinicians (with more than 10 years of experience) using the nominal group technique [31]. Structured interviews consisted of several items and prioritization exercises, each one investigating potential impacts related to the use of biologic drugs in the context of CRSwNP proposed to clinicians. The experts were asked to estimate the impact of the alternatives for each item through a 7-level Likert scale, validated for HTA investigation for the non-clinical domains [32]. For the conduction of research activities presenting a multidimensional nature, the definition of the adequate sample size is not applicable, as it is for randomized clinical trials or observational studies. Otherwise, the sample size is thus based on the definition of a convenient sample [33]. On the one hand, concerning the definition of qualitative information, by means of professionals’ perceptions retrieval, different hospital stakeholders involved in CRSwNP patients’ management were enrolled, to be representative of Italian hospitals’ context, thus guaranteeing the replicability of the qualitative results, in line with the literature evidence on the topic with regard to HTA analysis [33].

It is important to underline that the interviews were conducted at a time when neither omalizumab nor mepolizumab had been granted reimbursement in Italy yet, so Italian ENT expertise in using these drugs was not directly related to the treatment of CRSwNP but was associated with the observation of patients who were being treated with these drugs for asthma, evaluating the comparative potential impacts concerning all the non-clinical domains.

The dimensions investigated in the HTA analysis are the following.

- Relevance of the pathology: assessed through structured interviews and narrative literature analysis; the target population was quantified, as previously described.
- Safety: assessed by collecting adverse events rate following the use of the selected alternatives and stratified per reference treatment. Occurrence rates were gathered from selected studies, validated with the declared scales, and preferring primary evidence such as RCTs. Subsequently, this dimension was evaluated from an economic perspective, assessing the economic resources needed to manage healthcare issues related to adverse events and complications. Safety was also explored through structured interviews to gather expert perceptions involved in patient management and treatments.
- Efficacy: based on literature evidence [14], the 12-month improvement in key outcomes (SNOT-22, NPS, VAS, and UPSIT) for each treatment were compared. These parameters were calculated as the difference between the baseline values of the clinical scales and the final values, all divided by the baseline values. The reduction in the need for rescue surgery or OCS treatment was also considered, based on RCTs evidence of biological treatments. Expert perceptions regarding key outcomes were obtained through structured interviews.
- Equity of access dimension: assessing the ability of alternatives to increase access to care for the population in needing treatment for nasal polyposis. It was assessed through structured interviews.
- Organizational impact: represents the dimension to study the acceptability and the hospital processes implications related to the introduction of new drugs and technologies, considering both short- and long-term (12-month and 36-month time horizons). This domain was assessed through structured interviews to understand healthcare professionals’ perceptions. Additionally, the initial organizational and economic effort that hospitals should undertake to introduce biological drugs into clinical practice was estimated (both 12-month and 36-month time horizons).
- Social aspects: devoted to collecting patients’ points of view, assessed through structured interviews to gather healthcare personnel perceptions on the patients’ conditions.
- Legal aspects: factors influencing the regulation and introduction of the therapeutic alternatives, assessed through the structured interviews.
- Economic impact: conducted from the perspective of the National Healthcare System, considering three quantitative approaches derived from the health economics studies: activity-based costing and cost-effectiveness approaches (with two different measures) [34,35].

For the economic dimension, evaluated from a quantitative perspective, specific methodological information is provided below.

- Activity-based costing: the clinical pathway of CRSwNP encompassed several cost items.
  (1) Follow-up visits: the number of follow-up visits was estimated through data collection within 4 hospitals, to establish the clinical practice with regard to this activity in the Italian context, and this information was validated by the other clinicians. ENTs indicated that CRSwNP patients are typically visited by a specialist around once every 2.5 months. The cost of each follow-up visit was retrieved from the National Outpatient Tariff valid for 2023 (Italian Ministry of Health, National tariffs of NHS services, 2023).
  (2) Cost for asthma exacerbation management: this cost component was included due to its prevalent occurrence as a CRSwNP comorbidity. The proportion of patients with comorbid severe asthma was obtained from literature sources [7,36]. The rate of asthma exacerbation in these patients was derived by the literature evidence investigation [37] and multiplied by the cost of a single event (considering Italian DRG 097—Bronchitis and asthma, age > 17 years without CC, valid for the 2023).
  (3) Adverse events: the financial implications associated with adverse events management were derived by multiplying the treatment costs for each potential adverse event by its documented probability of occurrence across different alternatives, based on existing literature [15,16,17]. Incidence rates of adverse events for biologics were extrapolated from metrics documented in RCTs [15,16,17]. To ensure the validity of these metrics, expert consultants were asked to validate them through specific scales.
  (4) Risk of resorting to FESS (Functional Endoscopic Sinus Surgery) or OCS: in cases where biological drugs are ineffective, patients may need corticosteroids or surgery. Occurrence rates for these cases were derived from RCTs [15,16,17] and valued, with their weighted average costs calculated. The cost of treating a patient with FESS was valued by DRG 054 (National Tariff Schedule, Italian NHS perspective), and the cost of OCS treatment was based on dosage, frequency of intake cycles, and cost per package [38], multiplied by the probability of needing rescue OCS treatment.
- Cost-effectiveness measures were calculated considering both cost per responder (CPR) and cost per percentage of improvement. CPR is a methodological indicator that delineates [39] the economic expenditure associated with achieving a positive treatment response in therapeutic and medical interventions. Methodologically, the CPR is obtained by dividing the total expenditure incurred during a specific treatment pathway by the number of individuals who exhibit a favorable response, mathematically expressed as follows:
  $$\mathrm{CPR}=\frac{\mathrm{Total}\;\mathrm{cost}\;\mathrm{of}\;\mathrm{intervention}}{\mathrm{Number}\;\mathrm{of}\;\mathrm{responders}}$$
  The ‘total cost’ encompasses all management costs related to the intervention and included in the activity-based costing approach, multiplied per the number of treated patients. The ‘number of responders’ refers to patients exhibiting clinically meaningful improvement in the Nasal Polyp Score (NPS), defined as an improvement greater than 1 point [13]. Retrieval of the number of responders for each treatment was based on literature evidence [35]. This analytical approach provides a pragmatic insight, facilitating resource allocation by juxtaposing the economic and clinical dimensions of healthcare interventions, thereby guiding stakeholders in informed decision-making processes.
- Additionally, the cost per percentage of improvement in the main clinical scales, as outlined in EUnetHTA Core Model, examines the percentage improvement in CRSwNP’s clinical rating scales in relation to treatment costs. This method is well-suited for the technologies under investigation, due to their diverse efficacy profiles and cost structures. It helps determine the average cost incurred to achieve a “unit of effectiveness”, calculated by dividing the average cost of the technology (previously computed by time-driven activity-based costing) to its effectiveness parameter. The effectiveness parameters considered are the values of SNOT-22, NPS, VAS, and UPSIT.

## 3. Results 

### 3.1. Literature Review

The narrative literature evidence research started with the formulation of a PICO framework and the definition of a search string tailored for insertion into PubMed and Scopus databases. Utilizing the PRISMA diagram [40], the obtained results underwent a screened process, resulting in the selection of 21 papers deemed more relevant for this study.

**Figure 1:**
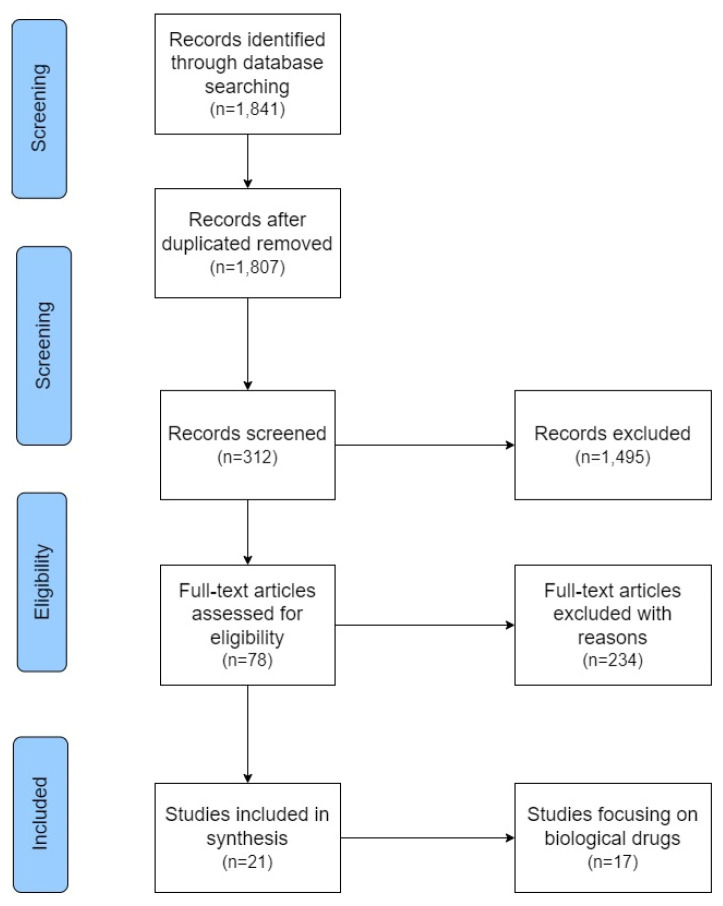
PRISMA flowchart.

The PRISMA diagram illustrating the conducted literature search is presented below (Figure 1).

The assessment of these papers’ quality was performed, yielding an average score of 4.7 out of 7 using the JADAD scale. Meanwhile, the evaluation based on the Newcastle–Ottawa Scale resulted in an average score of 5.3 out of 9. This indicates an overall acceptable quality of the literature references retrieved to achieve the study’s objectives.

### 3.2. Target Population

**Figure 2:**
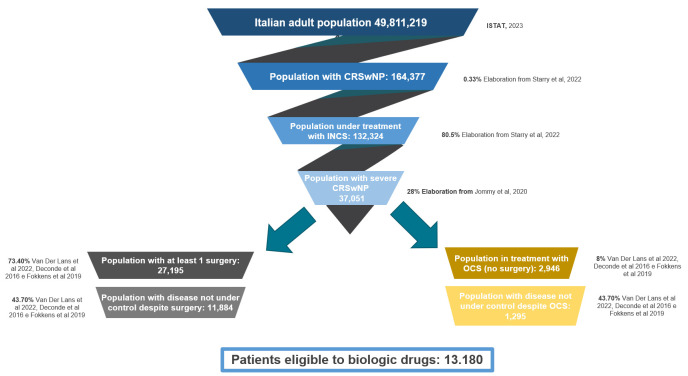
Population funnel, elaboration by authors [1,22,23,41,42].

The estimated number of patients with uncontrolled severe CRSwNP despite surgery and OCS was 11,884, while the number of patients uncontrolled despite only OCS treatment was 1,295. Thus, as a result of the evidence identified, a population of 13,180 patients were determined to be eligible for treatment. Potentially, these patients could benefit from biological drug treatments for type 2 inflammation disorders. Graphical representation is provided in Figure 2.

### 3.3. Safety

Adverse events related to each treatment are listed below in Table 1, together with their rate of occurrence [15,16,17]. It is important to underline that occurrence rates listed below are derived from a metanalysis based on a III-phase RCT, which presents slight differences in inclusion criteria.

A comprehensive analysis of adverse events drawn from various RCTs reveals discrepancies among several therapeutic options, notably, dupilumab, omalizumab, and mepolizumab. Asthma exacerbation demonstrates varying occurrence rates: dupilumab at 2% [16], omalizumab at 3.7% [15], and mepolizumab at 2% [17]. Moreover, differences emerged in headache incidence, with dupilumab at 7%, omalizumab at 8.1%, and mepolizumab, notably higher, at 18%.

As part of the safety analysis, healthcare professionals’ perceptions were qualitatively assessed through structured and validated questionnaires.

**Figure 3:**
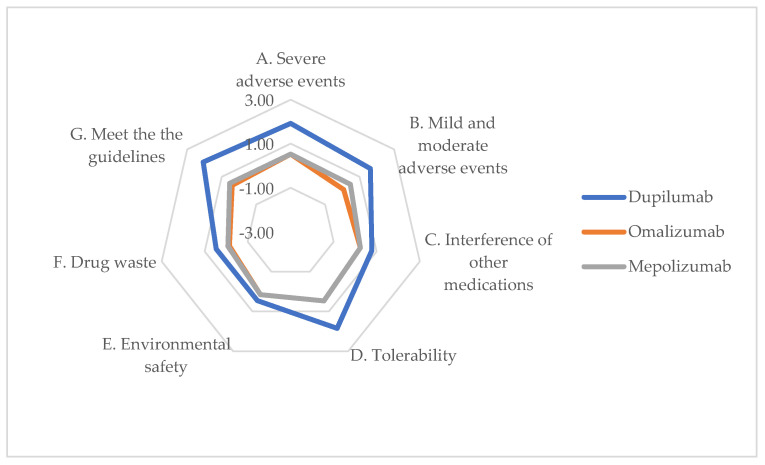
ENTs’ perceptions on safety of alternatives (chart).

Table 2 presents the average scores for each questionnaire item, with an additional graphical representation in Figure 3.

Dupilumab demonstrates a notably positive impact on both severe and mild-to-moderate adverse events (Items A and B). Conversely, Item E (Impact of alternatives on environmental safety within the hospital environment) exhibited not-significant deviation from the value 0, indicating that the alternatives do not notably affect the environment. Moreover, dupilumab also scored favorably in terms of general safety and tolerability (Item D, Degree of General Safety and Tolerability of Alternatives). This stands out as a primary advantage that biologic drugs offer over traditional treatments: prolonged usage of corticosteroids or multiple surgical interventions may lead to various healthcare issues. Respondents concurred that assessing the impact on safety might necessitate patient evaluation over two distinct time frames (at 1 month and 6 months).

### 3.4. Efficacy

Analysis of the literature allowed estimation of the improvement in primary clinical outcomes following treatment with various therapeutic alternatives [15]. Table 3 displays the baseline values of primary outcomes alongside the values at the end of treatment [14]. The outcomes come from a published metanalysis comparing the three biologics through a GRADE methodology.

Below, Table 4 is displaying the improvement in the primary clinical outcomes for CRSwNP, as detailed in the methodology section.

The likelihood of needing rescue surgery or OCS treatment was determined based on the same studies. The results are presented in Table 5, indicating that dupilumab demonstrates a more favorable efficacy profile to other available alternatives [15,16,17].

**Figure 4:**
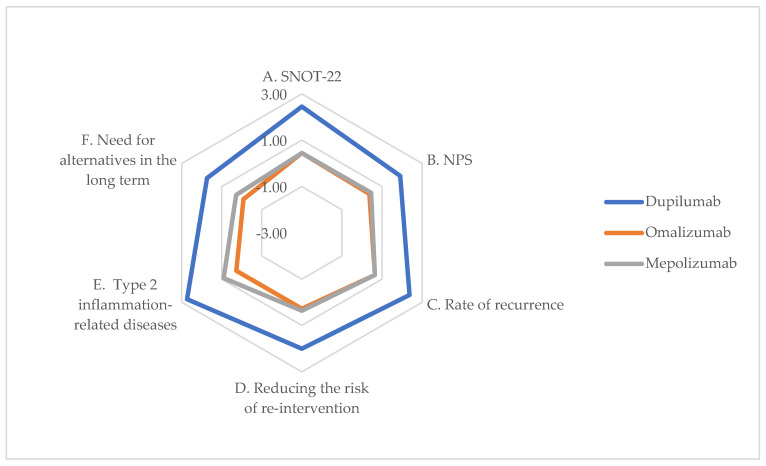
ENTs’ perceptions of efficacy of alternatives (chart).

As with the safety dimension, the efficacy of the alternatives was qualitatively assessed using a structured questionnaire administrated to ENT specialists. The mean scores for each questionnaire item are in Table 6, accompanied by a graphical presentation in Figure 4.

Based on professionals’ perceptions, biologic drugs maintain positive values across all investigated items. Dupilumab demonstrates a notably positive perceived impact, particularly in items of improving patient-reported outcomes, such as the SNOT Scale 22 (Item A), compared to the other biologic alternatives. Its efficacy in managing type 2 inflammation and relapse rate is highly regarded by both patients and clinicians. The interviewed clinicians agreed that, similar to safety assessment, evaluating the patient at two distinct time points (1 month and 6 months) might be necessary to gauge efficacy impact.

### 3.5. Equity Dimension

**Figure 5:**
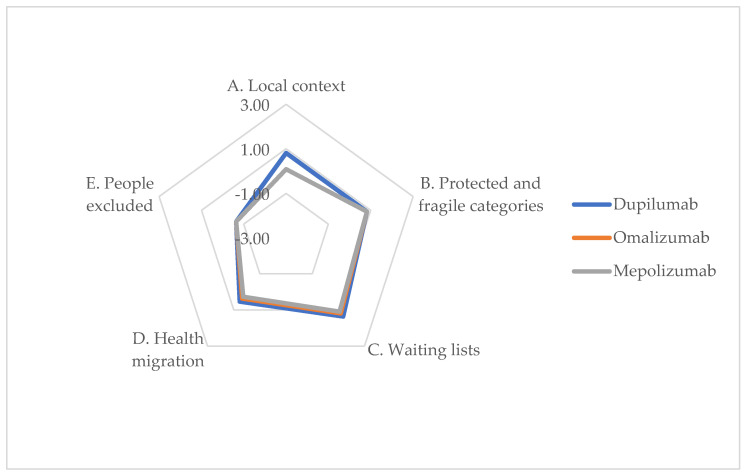
ENTs’ perceptions of impact on equity dimension (chart).

The results related to the dimension of equity of access to treatment are illustrated in Table 7, along with an accompanying graph in Figure 5.

Concerning the Potential Impact of Alternatives on Waiting Lists (Item C), respondents indicated that while follow-up visits may increase, surgical waiting lists could potentially decrease, especially in the long-term, due to a greater use of biologic drugs. However, the accessibility of biologic therapies within the territory, particularly for omalizumab and mepolizumab, was affected due to their non-reimbursable status, in the Italian NHS for CRSwNP, at the time the interviews were conducted. The potential reduction in surgical waiting lists could profoundly affect healthcare systems, suggesting a re-evaluation of current resource allocation to accommodate the integration of biologic therapies. Additionally, the phenomenon of health migration, indicated by the perceptions of accessibility and ability of alternatives to induce healthcare migration (Items A and D), raises concern about the consistency of care and the necessity to mitigate this through more uniform healthcare policies. Such considerations are critical in shaping future strategies to enhance the delivery of personalized medicine, ensuring equitable access to the most effective treatments for all patients, regardless of their socioeconomic status or geographical location. The potential impact on waiting lists (Item C) also suggests that, although follow-up visits may increase, the overall efficiency of healthcare delivery could improve, especially over the long term. These aspects, taken individually, seem to have non-differential impact among the alternatives, but in the overall size assessment, they are in favor of the biologic that, at the time of the study, has greater accessibility, namely, dupilumab.

### 3.6. Social Dimension

**Figure 6:**
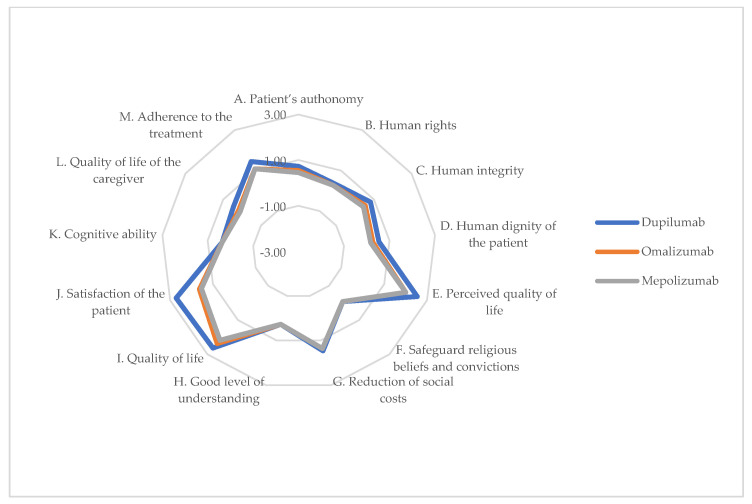
ENTs’ perceptions of impact on social dimension of alternatives (chart).

The perceptions collected from the perspectives of the healthcare professionals regarding the three biologic drugs were substantially overlapping and scored significantly better on the items listed in Table 8. Presently, biologic drugs do not fully guarantee patient autonomy (Item A) as patients are required to pick up the drug from the hospital facility where they receive care. Patients using biologic drugs perceive an improved quality of life compared to other treatments. Specifically, dupilumab is highly regarded for patient satisfaction (Item J), as it effectively manages the disease and its symptoms, reducing the need for therapeutic changes over time. Regarding therapeutic adherence (Item M), patients undergoing treatment during this period were carefully selected and monitored, resulting in high adherence levels. However, some respondents expressed minimal concerns that long-term use may lead to poor adherence. This concern emphasizes the importance of continual patient engagement and education to maintain treatment effectiveness. Graphical representation is provided in Figure 6.

The overall positive perceptions collected regarding the biologics highlight their ability to enhance the quality of life and satisfaction. This underscores their potential to address not only the clinical aspects of CRSwNP, but also the broader social implications of living with this chronic condition.

### 3.7. Legal Dimension

**Figure 7:**
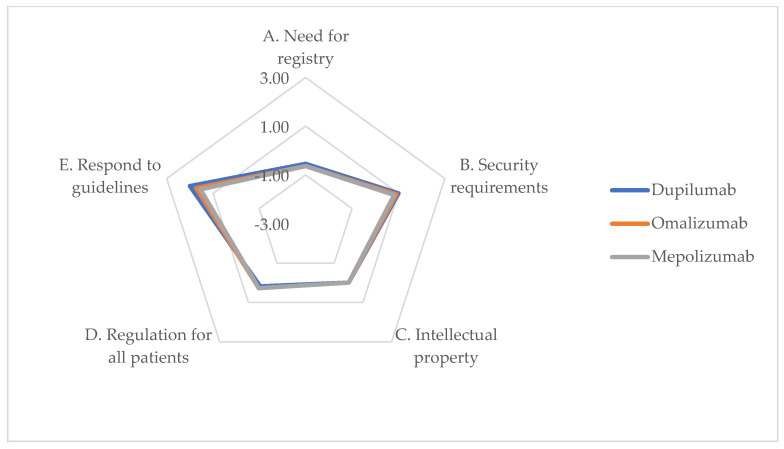
ENTs’ perceptions of impact on legal dimension of alternatives (chart).

This analysis revealed a positive legal impact of biologic drugs, particularly in terms of Satisfaction of Safety Requirements (Item B) and Ability to Respond to Guideline Guidance (Item E). Generally, the considered alternatives share similar ratings for this dimension. The impressive performance of biologics in meeting safety standards and adhering to guidelines emphasizes their compliance with existing legal and regulatory frameworks. This underscores the importance of continuous adherence as these treatments become more integrated into clinical practice. Item list and responses are reported in Table 9, and graphical representation is provided in Figure 7.

### 3.8. Organizational Impact

**Figure 8:**
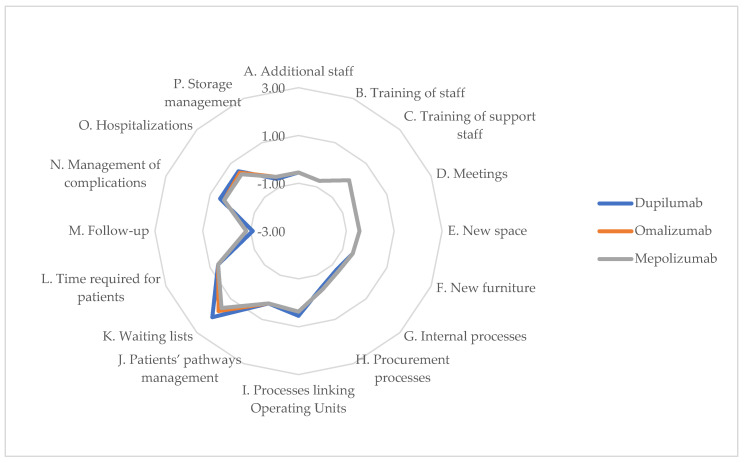
ENTs’ perceptions of impact on organizational dimension of alternatives (12 months—chart).

**Figure 9:**
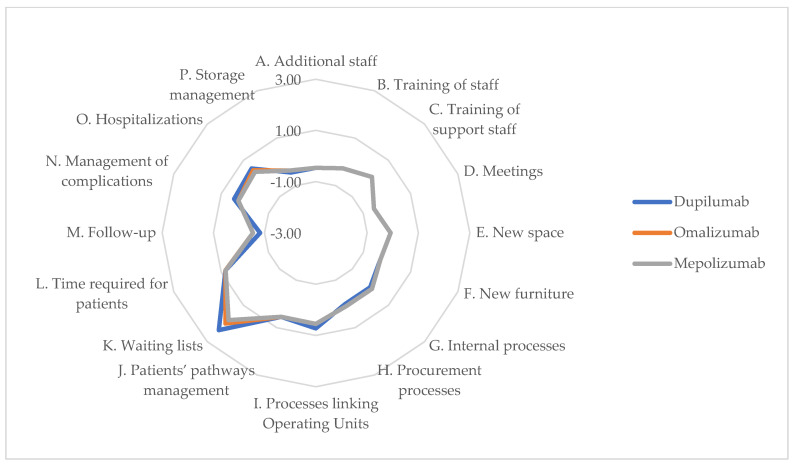
ENTs’ perceptions of impact on organizational dimension of alternatives (36 months—chart).

Analyzing the organizational impact, the values assigned by experts to the three biological drugs used in the short term were lower compared to the long-term results. This indicates that, after an initial adaptation period, organizations are likely to adopt these technologies, benefiting from the learning curve and reducing the overall impact on hospitals and healthcare personnel. Positive values were observed regarding items corresponding to the efficiency of surgical waiting lists and overall processes. While initial investments in training and infrastructure may pose challenges, the long-term benefits, like improved surgical waiting lists, underscore the organizational commitment to adopting innovative treatments. Items and perceptions for short and long term are reported in Table 10, while short term chart and long term chart are represented, respectively, in Figure 8 and Figure 9.

In the short term, the implementation of these biologics in clinical practice could show significant improvements from the perspective of both internal and external processes, within and outside the operating unit, as well as in overall patient management, notably in terms of the Impact on Surgical Waiting Lists (Item K). The data suggest a shift towards more efficient patient management, which could reduce the reliance on surgical interventions. 

In the long term (36 months after introduction), there were improvements seen with higher scores on multiple items. Particularly, there were increases in internal efficiency for both biologics and corticosteroid treatments, shown by the shift in Item G (Impact on Internal Processes of the Operating Unit) from negative short-term scores to positive long-term ones. These trends illustrate a positive adaptation by healthcare organizations in integrating new biologic treatments into their service offerings, possibly leading to more sustainable patient management strategies. Additionally, while an increase in follow-up visits is expected, this could correspond to a decrease in operating rooms utilization, indicating more effective disease control and therapy as patients receive more frequent monitoring. 

### 3.9. Economic Impact

1. Activity-Based Costing

The results of activity-based costing showed differences among the three treatments, based on the cost items reported and studied.

Recurrence of FESS Surgery: Recurrence of FESS surgery has a direct cost of EUR 91.38 when treated with dupilumab. Compared with this, omalizumab and mepolizumab show a cost increase of 65.57% and 46.10%, respectively. 

Need for OCS (Oral Corticosteroids): Dupilumab appears to be associated with increased costs for OCS need (EUR 1.92), but to a lesser extent than the other two biologics (omalizumab and mepolizumab have a higher cost of 47.68% and 53.06%, respectively). 

Follow-up: All three biologic treatments have an identical cost for the follow-up management (EUR 412.30), since there is no difference in the number of follow-up visits needed for these treatments, an aspect related to the correct pathology management.

Adverse events: The cost management of adverse events is 51.48 EUR for dupilumab. Compared with dupilumab, omalizumab and mepolizumab show an increase in cost of 23.55% and 18.67%, suggesting that dupilumab may have a slightly better safety profile management, with less costs associated with treating adverse events.

Management of asthma exacerbation: Dupilumab shows a cost of EUR 66.77 for the management of asthma exacerbation, while omalizumab and mepolizumab have a cost increase of 21.95% and 27.27%, respectively. Dupilumab is more beneficial in managing asthma exacerbations associated with CRSwNP.

### 3.10. Cost per Responder

The cost per responder (CPR) analysis provides an advanced perspective in assessing the financial implications of biologic therapies. This metric represents a cost-effectiveness measure that specifically delineates the point where clinical success aligns with financial investment. By computing the cost invested for each patient who achieves a significant clinical response, the CPR analysis provides a multifaceted viewpoint crucial for a comprehensive therapeutic efficacy assessment, complementing other proposed cost-effectiveness measures. It underscores the delicate balance between financial prudence and the pursuit of the best possible patient outcome, which is pivotal information for healthcare legislation. In the context of personalized medicine, where treatments are tailored to individual needs and responses, the CPR assumes heightened significance. It ensures that economic decisions made by healthcare providers do not overlook the ultimate aim of medicine: effectively alleviating disease burden from the patient’s perspective. Patients were considered responders if they showed an improvement > 1 point in the NPS score after 1 year of treatment. Table 11 shows the response rate for each treatment, together with cost per responder values.

In this analysis, dupilumab is the most favorable biologic drug, and its cost per responder is 43.16% and 55.41% lower than, respectively, omalizumab and mepolizumab.

2. Cost per percentage of improvement in key outcomes

Table 12 illustrates the cost to the Italian National Healthcare System for each of the three biological treatments to achieve the same level of effectiveness.

As can be seen in Table 12, dupilumab represents the option that allows patients to achieve equivalent efficacy, according to major clinical scales, but with a lower economic impact to the Healthcare System.

## 4. Discussion

For the first time, this study has offered a comprehensive understanding of the effects of biological drugs on CRSwNP patients. The economic and organizational effects are a critical emphasis of this investigation, which also highlights the place of dupilumab among biological alternatives. 

The current literature on the subject, in the few cases where a comparative evaluation between biological drugs has been conducted, tends to limit itself to comparing their clinical efficacy, neglecting other relevant factors [43]. Focusing solely on the efficacy analysis presented in these studies, it is evident that the results are consistent with the qualitative findings of our work, based on interviews with 17 ENT experts, whose practical experience highlights the superiority of dupilumab [20,44]. Although our study focuses on the Italian context, the efficacy results are also in line with those from research conducted in other settings, both within and outside the European Union [45,46,47,48,49].

It shows a good trade-off between the costs incurred by the Italian National Healthcare Service and the efficiency of the therapeutic results. This balance is attained as dupilumab, despite requiring a higher initial investment than its comparators, shows superior efficacy. This is evident in its cost-effectiveness, both in terms of cost per responder and the cost per percentage improvement in critical outcomes. The assessment of treatment costs in this study was achieved through the precise measures of resources used in the patient treatment pathway. The efficacy outcomes comparison relied on indirect treatment comparisons and meta-analyses, made with the purpose of comparing different populations. However, future research would benefit from head-to-head comparative studies between treatments, enhancing the precision of these analyses. A distinctive aspect of the conducted study is the employment of a quantitative approach to investigate the comparative economic elements of three biological agents, revealing a substantial overlap in resource utilization across the alternatives. The primary distinctions, however, pertain to the enhancements in the efficacy and safety profiles. This analysis delineates dupilumab as the optimal choice in terms of cost per responder (CPR) and cost per percentage of improvement in key outcomes, thereby optimizing the patient care pathway. Upon examining the results pertaining to non-clinical domains, a congruence in professional perceptions regarding the different alternatives becomes apparent. There is a slight, statistically significant preference for dupilumab, particularly with respect to aspects of accessibility and social impact. The 12-month time horizon used for cost estimation in this study is a limitation, as it fails to account for potential costs that the NHS may incur in later years. The recurrence of the disease is a common dynamic, and the surgical option and outcome are closely related to the time horizon. For instance, of the patients treated with surgery, a significant 17.88% need to undergo at least two interventions within a 24-month period, but this percentage is almost null in the first 12 months [31]. For this reason, the present evaluation could be considered a conservative analysis not including all the long-term benefits related to the biologic treatments. Despite the outlined limitations, this work establishes, for the first time, an attempt to estimate the total process costs for the use of biological drugs in this specific pathology setting, particularly at a historical moment when three active ingredients have received approval for use. In the current climate for the NHS, evaluating both the advantages and disadvantages of selecting one therapeutic option over another becomes essential, aiding healthcare professionals, decision-makers, and those in charge of managing pharmaceutical spending in navigating towards optimizing treatment while maintaining a keen awareness of the consequences. The findings on safety highlight the importance of personalized treatment strategies, tailoring therapies to individual patient profiles to achieve the best outcomes. Surgical interventions, though associated with lower rates of adverse events, could lead to significant patient complications and present complex management challenges for healthcare facilities. 

Additionally, a novel aspect investigated by this study is the incorporation of non-clinical domains, made possible by utilizing the Core Model of the European Network for Health Technology Assessment (EUnetHTA). The analysis revealed a general alignment among the three treatment alternatives, with dupilumab consistently standing out due to its superior profile in all evaluated aspects. As previously stated, the analysis was conducted through the collection of perceptions from professionals in the field. It is important to note, however, that this analysis was based on the perceptions of professionals who had not directly treated CRSwNP patients with omalizumab and mepolizumab. Nevertheless, this work represents a multidimensional approach in a clinical setting where no other evidence exists on these topics and dimensions, underlying the importance of collecting these perceptions to create a baseline expert opinion paving the way for future quantitative studies. Moreover, all HTA evaluations represent an estimation of potential impact, which will then need to be consolidated through the collection of real-world evidence or updated considering the alternative technologies lifecycle. The hope of this work is that it may be verified through comparative studies based on real-world data. This decision-making process should be informed by a balanced consideration of clinical effectiveness, safety, and economic impact. Future research aiming to close the current gap in the literature for comparative, longitudinal investigations of various treatment modalities over a significant time horizon may offer a more solid foundation for treatment decision-making and supporting policymakers’ decision processes.

### Limitations

This study integrates findings from a range of scientific articles worldwide to assess the efficacy of biological drugs in treating CRSwNP, which introduces a key limitation, that is, the applicability of these global insights to the Italian population. Notably, the research does not exclusively draw upon data from Italian-based studies. This broad approach overlooks the geographical variability in CRSwNP endotypes, which can significantly influence treatment outcomes. Given that endotypes of CRSwNP have distinct distributions across different regions, the results from international studies might not accurately reflect the situation in Italy.

Additionally, the diversity in methodologies and study designs among the sourced articles could affect the relevance of their conclusions for the Italian context. This variation underscores the challenge in directly applying international findings to Italy’s unique demographic and epidemiological landscape.

As previously stated, a part of this study was conducted thanks to interviews with 17 ENTs who did not have direct experience with all of the biological drugs under assessment in this study. Finally, the last limitation is the 12-month time horizon that was chosen for the analyses: this choice was made because of the availability of data in the literature, but, to be more exhaustive, a wider time lapse would be more appropriate to assess the effect of a chronic disease.

## 5. Conclusions

Although the purchase price of biologic drugs is significantly higher than that of usual care alternatives, the potential to reduce the economic impact related to adverse events and comorbidities could offset part of the initial cost. However, this could be a deterrent to their adoption by hospitals, particularly public ones for budget reasons. Hospitals must consider long-term consequences for patients in terms of safety, efficacy, and economic benefits, rather than choosing short-term economical therapeutic alternatives. Decision-makers should employ a holistic approach to treatment selection, balancing immediate costs with long-term patient outcomes. The recent introduction of biological alternatives limits the availability of long-term data, making it challenging to estimate the impacts of dupilumab, omalizumab, and mepolizumab over several years. Therefore, the choice between different biological agents should be driven not only by cost but also by their efficacy, especially in alleviating symptoms like anosmia, which significantly affect patient health but are hard to quantify in cost terms. This gap in the literature underscores the need for more extensive research to fully understand these biologics’ long-term effects. Future studies—both comparative and longitudinal—will be essential in directing healthcare providers and policymakers toward CRSwNP treatment approaches that are more viable and efficient.

## Figures and Tables

**Table 1:** Adverse events rates.

Adverse events	Occurrence Rate with Dupilumab	Occurrence Rate with Omalizumab	Occurrence Rate with Mepolizumab
Asthma exacerbation	2.00%	3.70%	2.00%
Headache	7.00%	8.10%	18.00%
Injection site reactions	6.00%	5.20%	0.00%
Nasopharyngitis	13.00%	5.90%	25.00%
Eosinophilia	1.00%	0.00%	0.00%
Arthralgia	0.00%	3.00%	6.00%
Back pain	0.00%	3.00%	7.00%
Fatigue	0.00%	3.00%	0.00%
Epistaxis	6.00%	3.00%	8.00%
Rhinitis	0.00%	3.00%	0.00%
Sinusitis	0.00%	3.00%	5.00%
Oropharyngeal pain	0.00%	0.00%	8.00%
Respiratory tract infection	0.00%	0.00%	4.00%
Bronchitis	0.00%	0.00%	5.00%
Cough	0.00%	0.00%	3.00%
Otitis	0.00%	0.00%	2.00%

**Table 2:** ENTs’ perceptions on safety of alternatives.

Items	Dupilumab	Omalizumab	Mepolizumab	*p*-Value
Impact of alternatives on the development of severe adverse events (SAEs)	1.92	1.08	0.92	0.000
B. Impact of alternatives on the development of mild and moderate adverse events (e.g., weight gain, hypertrichosis, steroid diabetes, ulcers, bleeding, osteoporosis, etc...)	1.62	0.85	0.77	0.000
C. Impact of alternatives on the development of issues related to the potential degree of interference of other medications with the therapy	0.77	0.62	0.62	0.000
D. Degree of general safety and tolerability of alternatives	1.85	1.15	1.08	0.000
E. Impact of alternatives on environmental safety (hospital environment)	0.46	0.38	0.38	0.767
F. Impact of alternatives on drug waste generation	0.46	0.38	0.38	0.001
G. Ability of the alternatives to meet the guidance provided by the guidelines	2.08	1.46	1.38	0.001
Average	1.31	0.85	0.79	0.000714

**Table 3:** Clinical scale parameters, baseline and endpoint.

Efficacy Parameters	SNOT-22	NPS	VAS	UPSIT
Baseline	50.11	5.94	6.84	14.04
dupilumab	30.20	3.90	3.59	10.96
omalizumab	34.02	4.85	4.75	3.75
mepolizumab	37.22	4.88	5.02	6.13

**Table 4:** Efficacy improvement (SNOT-22, NPS, VAS, UPSIT).

Efficacy Parameters	SNOT-22	NPS	VAS	UPSIT
dupilumab	−39.73%	−34.34%	−47.51%	78.06%
omalizumab	−32.11%	−18.35%	−30.56%	26.71%
mepolizumab	−25.72%	−17.85%	−26.61%	43.66%

**Table 5:** Probability of reduction in rescue OCS or FESS.

Reduction in Resort Standard Treatment	Dupilumab	Omalizumab	Mepolizumab
Probability to resort rescue OCS treatment	−21.73%	−12.46%	−10.23%
Probability to resort rescue FESS	−16.35%	−7.40%	−12.33%

**Table 6:** ENTs’ perceptions of efficacy of alternatives.

Item	Dupilumab	Omalizumab	Mepolizumab	*p*-Value
Impact of alternatives on improvement of patient-reported outcomes (SNOT-22 Scale)	2.45	1.00	0.80	0.000
B. Impact of alternatives on the improvement of polyp size (NPS score)	1.91	1.00	0.80	0.001
C. Impact of alternatives on the rate of recurrence of signs and symptoms of pathology	2.36	1.20	1.00	0.000
D. Impact of alternatives on reducing the risk of re-intervention	2.00	0.90	0.70	0.000
E. Impact of alternatives on perceived improvement on Type 2 inflammation-related diseases (e.g., atopic dermatitis, asthma, etc...)	2.73	1.64	1.73	0.000
F. Impact of alternatives on the need for therapeutic alternatives in the long term	1.73	0.60	0.20	0.000
Average	2.20	1.06	0.87	0.000623

**Table 7:** ENTs’ perceptions of impact on equity dimension.

Item	Dupilumab	Omalizumab	Mepolizumab	*p*-Value
Accessibility of alternatives in the territory and in the local context of reference	0.82	0.09	0.09	0.99
B. Accessibility of alternatives to protected and fragile categories	0.82	0.82	0.82	0.950
C. Potential impact of alternatives on the waiting lists	1.36	1.18	1.09	0.531
D. Ability of alternatives to generate phenomena of health migration in case of use	0.55	0.36	0.27	0.945
E. Existence of factors that could prevent a group or certain people from benefiting from the indicated alternatives	−0.64	−0.64	−0.64	0.979
Average	0.58	0.36	0.33	0.000011

**Table 8:** ENTs’ perceptions of impact on social dimension of alternatives.

Item	Dupilumab	Omalizumab	Mepolizumab	*p*-Value
Ability of alternatives to safeguard patient’s autonomy	0.73	0.55	0.45	0.945
B. Ability of alternatives to safeguard human rights	0.27	0.55	0.55	0.913
C. Capacity of the alternatives to safeguard human integrity	0.82	0.55	0.45	0.683
D. Capacity of alternatives to ensure human dignity of the patient	0.18	0.45	0.27	0.839
E. Impact of alternatives on comorbidities related to Type 2 inflammation in terms of quality of life (e.g., atopic dermatitis, asthma, etc.)	2.00	0.82	0.36	0.000
F. Ability of alternatives to safeguard religious beliefs and convictions	−0.09	0.27	0.00	0.843
G. Impact of alternatives on the reduction in social costs, understood as those costs directly borne by the patient or his or her family, with reference to the treatment of the disease (cost of transportation, lack of productivity of the patient or accompanying family members, direct purchase of drugs by patients, etc...)	1.65	−1.91	−0.82	0.000
H. Patients and the general public may have a good level of understanding of the alternatives	0.27	1.09	1.18	0.469
I. Impact of the alternatives on the quality of life of the patient	2.89	0.36	0.55	0.000
J. Impact of alternatives on the satisfaction of the patient	1.55	0.91	1.00	0.002
K. Impact of alternatives on the cognitive ability of the patient	0.36	−0.18	0.00	0.383
L. Impact of alternatives on the quality of life of the caregiver	0.09	0.27	−0.09	0.837
M. Impact of alternatives on adherence to the treatment pathway by patients	1.09	−1.09	0.27	0.000
Average	0.86	0.20	0.24	0.000

**Table 9:** ENTs’ perceptions of impact on legal dimension of alternatives.

Item	Dupilumab	Omalizumab	Mepolizumab	*p*-Value
Need for registry entry at national/European level	−0.55	−0.64	−0.64	0.828
B. Fulfillment of required security requirements	1.00	0.91	0.82	0.730
C. Infringement of intellectual property rights	0.00	0.00	0.00	-
D. Legislation covers regulation of alternatives for all categories of patients who may benefit, in accordance with specific indications	0.18	0.27	0.27	0.959
E. Ability to respond to indications provided by guidelines	2.00	1.73	1.15	0.094
Average	0.53	0.45	0.32	0.000

**Table 10:** ENTs’ perceptions of impact on organizational dimension of alternatives (12 and 36 months).

Item	Dupilumab (Short-Term)	Dupilumab (Long-Term)	Omalizumab (Short-Term)	Omalizumab (Long-Term)	Mepolizumab (Short-Term)	Mepolizumab (Long-Term)	*p*-Value (Short-Term)	*p*-Value (Long-Term)
Use of alternatives requires additional staff	−0.55	−0.45	−0.55	−0.45	−0.55	−0.45	0.091	0.204
B. Use of alternatives requires training of staff responsible for the procedure	−0.73	−0.27	−0.73	−0.27	−0.73	−0.27	0.627	0.845
C. Use of the alternatives requires training of support staff involved in the processes involved in the use of the specific alternative (from a care or surgical perspective)	0.00	0.09	0.00	0.09	0.00	0.09	0.964	0.995
D. Use of alternatives requires meetings for the management of the specific technological alternative	−0.45	−0.55	−0.45	−0.55	−0.45	−0.55	0.622	0.370
E. Utilization of alternatives requires new space needed for management of the technological alternative	−0.45	−0.09	−0.45	−0.09	−0.45	−0.09	0.801	0.997
F. Use of alternatives requires new furniture needed to manage technological alternatives	−0.55	−0.27	−0.55	−0.27	−0.55	−0.27	0.200	0.586
G. Impact of the alternatives on the internal processes of the relevant Operating Unit	−0.73	0.00	−0.64	0.09	−0.64	0.09	0.338	0.989
H. Impact of alternatives on the procurement processes of the Company	−0.45	0.00	−0.36	0.09	−0.36	0.09	0.885	0.991
I. Impact of alternatives on processes linking Operating Units	0.55	0.73	0.36	0.55	0.36	0.55	0.899	0.880
J. Impact of alternatives on patients’ pathways for both medical and surgical patient management	0.27	0.55	0.27	0.55	0.27	0.55	0.521	0.289
K. Impact of alternatives on the improvement of surgical waiting lists	2.09	2.36	1.73	2.00	1.55	1.82	0.073	0.000
L. Impact of alternatives on the average time required for the patient’s treatment pathway	0.64	0.82	0.64	0.82	0.64	0.82	0.932	0.690
M. Impact of alternatives on patient management with regard to follow-up visits	−1.09	−0.82	−0.82	−0.55	−0.82	−0.55	0.280	0.701
N. Impact of alternatives in the management of the patient with regard to the management of complications and adverse events	0.55	0.45	0.36	0.27	0.36	0.27	0.106	0.154
O. Impact of alternatives in patient management for what concerns hospitalizations	0.55	0.55	0.45	0.45	0.36	0.36	0.037	0.037
P. Impact of alternatives on storage management aspects of the same (e.g., refrigeration system)	−0.64	−0.45	−0.55	−0.36	−0.55	−0.36	0.042	0.093
Average	−0.06	0.16	−0.08	0.15	−0.10	0.13	0.000	0.000

**Table 11:** Response rates and cost per responder.

Treatment	Response Rate at Week 52	Cost Per Responder
dupilumab	67.0%	14,209.93 EUR
omalizumab	36.2%	24,999.32 EUR
mepolizumab	28.5%	31,863.78 EUR

**Table 12:** Cost per percentage of improvement in key outcomes.

Cost per Percentage of Improvement in Key Outcomes	SNOT-22	NPS	VAS	UPSIT
dupilumab	23,960.15 EUR	27,719.96 EUR	20,035.91 EUR	12,195.31 EUR
omalizumab	28,175.46 EUR	49,301.78 EUR	29,608.23 EUR	33,871.82 EUR
mepolizumab	35,319.75 EUR	50,912.76 EUR	34,145.29 EUR	20,809.07 EUR

## Data Availability

The data presented in this study are available on request from the corresponding author.

## Abbreviations

CRSwNP	Chronic Rhinosinusitis with Nasal Polyps
NMA	Network Meta-Analyses
ITC	Indirect Treatment Comparisons
HTA	Health Technology Assessment
OCS	Oral CorticoSteroids
INCS	Intra-Nasal CorticoSteroids
ISTAT	National Institute of Statistics
AIFA	Italian Drug Agency
SNOT-22 scale	Sino-Nasal Outcome Test 22 items
NPS scale	Nasal Polyp Score
VAS	Visual Analogue Scale
UPSIT scale	University of Pennsylvania Smell Identification Test
ENT	Ear, Nose and Throat specialist
CPR	Cost Per Responder
FESS	Functional Endoscopic Sinus Surgery
